# Predominance of enterotoxigenic *Escherichia coli* among ESBL/plasmid-mediated AmpC-producing strains isolated from diarrheic foals: a public health concern

**DOI:** 10.1186/s13028-024-00774-6

**Published:** 2024-10-03

**Authors:** Ahmed Samir, Khaled A. Abdel-Moein, Hala M. Zaher

**Affiliations:** 1 Department of Microbiology, Faculty of Veterinary Medicine, Cairo University, Cairo, Egypt; 2 Department of Zoonoses, Faculty of Veterinary Medicine, Cairo University, Cairo, Egypt

**Keywords:** Enterotoxigenic *Escherichia coli*, ESBL-/pAmpC, Foals, Public health

## Abstract

**Background:**

The upsurge of diarrheagenic *E*. *coli* pathotypes carrying extended-spectrum beta-lactamases (ESBLs)/plasmid-mediated AmpC β-lactamase (pAmpC) among animals constitutes an emerging threat for humans and animals. This study investigated the burden of ESBL-/pAmpC-producing diarrheagenic *E. coli* among diarrheic foals and its potential public health implications. Rectal swabs were collected from 80 diarrheic foals. These swabs were processed to isolate and identify ESBL/pAmpC-producing *E. coli* using a selective culture medium, biochemical tests, phenotypic identification, and molecular identification of ESBL- and pAmpC-encoding genes. Moreover, all ESBL-/pAmpC-producing *E. coli* isolates were examined for different virulence genes related to diarrheagenic *E. coli* pathotypes.

**Results:**

Out of 80 examined foals, 26 (32.5%) were confirmed as ESBL-/pAmpC-producing *E. coli,* of which 14 (17.5%) animals carried only ESBL-producing *E. coli*, whereas 12 (15%) animals possessed ESBL-pAmpC-producing *E. coli*. The only detected diarrheagenic pathotype was enterotoxigenic, encoded by the heat-stable enterotoxin gene (ST) with a prevalence rate of 80.8% (21/26). The ST gene was further characterized where STa, STb, and STa + STb were found in one, four, and 16 strains, respectively. Moreover, all enterotoxigenic *E. coli* (ETEC) isolates exhibited a multidrug-resistance pattern. The phylogenetic analysis of 3 obtained partial STb sequences revealed high genetic relatedness to ETEC isolates retrieved from humans, conferring such sequences' public health significance.

**Conclusions:**

These findings highlight that diarrheic foals could serve as a potential reservoir for multidrug-resistant ESBL-/pAmpC-producing enterotoxigenic *E. coli*.

## Background

*Escherichia coli* (*E. coli*) is a ubiquitous bacterium in the gastrointestinal tract of humans and animals, and some strains are pathogenic causing life-threatening infections in humans [1]. *E. coli* is a noteworthy microbe as it has developed resistance to several antimicrobials, which may be responsible for treatment failures in both humans and animals. Many antibiotic-resistance genes are acquired through horizontal gene transfer [2]. One of the essential mechanisms of antibiotic resistance is the production of extended-spectrum beta-lactamases (ESBLs), which represent one of the highest public health threats in hospital and community settings [3]. Apart from ESBLs, the production of plasmid-mediated AmpC β-lactamase (pAmpC) is another mechanism that confers resistance against penicillins, cephamycin, first- to third-generation cephalosporins, as well as beta-lactamase inhibitors [4]. Extended-spectrum beta-lactamase encoding genes are located on plasmids or chromosomes, which may provide resistance to broad-spectrum β-lactams, resulting in multi-drug resistance (MDR) with limited therapeutic options [5]. Recently, ESBL production has become a worrisome issue in the veterinary field, as there are subsequent reports of ESBL-producing *E. coli* in farm animals [6, 7] and companion animals [8, 9]. The risk of zoonotic transmission of ESBL-producing *E. coli* between horses and their owners is a public health concern [10, 11]. It is worth mentioning that antimicrobial resistance genes may co-exist with virulence determinants on the same plasmids in *E. coli* [12], where several diarrheagenic *E. coli* pathotypes have been identified: enterotoxigenic *E. coli* (ETEC), enteropathogenic *E. coli* (EPEC), enterohemorrhagic *E. coli* (EHEC), enteroaggregative *E. coli* (EAEC), and enteroinvasive *E. coli* (EIEC) strains [13]. The burden of virulent and ESBL-producing *E. coli* has been investigated in horses [9, 14, 15], while knowledge regarding the association between ESBL-producing *E. coli* and diarrheagenic pathotypes in foals is still scarce. ESBL-producing *E. coli* strains have been detected in foals with enteritis [16] and hospitalized neonatal foals [17]. Concerning diarrheagenic *E. coli*, one *E. coli* isolate obtained from diarrheic foals was positive for the STb and LT genes of enterotoxigenic *E. coli* [18]. However, in another study, no ETEC toxins (STa, STb, and LT) have been recognized in *E. coli* isolates recovered from diarrheic foals [19]. Therefore, the main objective of the current study was to investigate the prevalence of ESBL-/pAmpC-producing *E. coli* strains carrying virulence genes of diarrheagenic *E. coli* pathotypes among diarrheic foals and their public health implications.

## Methods

### Sample collection

This study was conducted at eight equine farms (50–100 horses per farm) in Giza governorate, Egypt. Ten diarrheic foals aged 1–3 months from each farm were selected based on experiencing bouts of watery diarrhea for 24 h or more. Diarrhea was accompanied with signs of colic, inappetence, weight loss and dehydration. All farms were similar regarding general animal management, hygiene, and facilities. The sample size was calculated depending on the previous estimated prevalence of ETEC among diarrheic foals (1.6%) from a prior study [18] as follows:

$$n = \frac{{z2x\hat{\user2{p}}(1 - \hat{\user2{p}})}}{{\user2{\varepsilon 2}}}$$

The parameters include the following: **N** is the population size, **z** is the z score corresponding to a 95% confidence interval (1.96), **ε** is the margin of error, and **p̂** is the population proportion. Rectal swabs were obtained from eighty diarrheic foals from August to December 2021. Sterilized cotton swabs were inserted into the rectum, placed in Cary-Blair transport medium tubes (HiMedia, India), and transferred in an ice box to the laboratory for immediate bacteriological examination.

### Isolation and identification of *E. coli*

Swabs were directly cultured on MacConkey agar (HiMedia, India) supplemented with cefotaxime (2 mg/L) (HiMedia, India) and incubated at 37 °C for 24 h. A single pure colony (pink in color) was picked up and subcultured on eosin methylene blue (EMB) agar (HiMedia, India) to obtain pure colonies. After that, *E. coli* was presumptively identified based on colonial appearance on EMB agar (green metallic sheen), Gram staining, and conventional biochemical tests. Isolates were then confirmed as *E. coli* using the RapID ONE system (Remel, USA). All confirmed isolates were inoculated into tubes containing 5 mL brain heart infusion broth (HiMedia, India) and incubated for 24 h at 37 °C. Each isolate was preserved with 20% glycerol at – 20 °C.

### Phenotypic identification of ESBL/AmpC-producing *E. coli*

All *E. coli* strains were subjected to ESBL phenotypic identification by the double-disk approximation test using both cefotaxime and ceftazidime, alone and in combination with clavulanic acid (ceftazidime-clavulanate (30/10 μg) and cefotaxime-clavulanate (30/10 μg)), according to CLSI guidelines [20]. Additionally, *E. coli* isolates were considered presumptive AmpC producers when resistant to cefotaxime and/or ceftazidime and cefoxitin [21].

### Molecular detection of ESBL- and pAmpC-encoding genes

DNA extraction was performed from presumptive ESBL/pAmpC-producing *E. coli* isolates via boiling [22]. Then, multiplex PCR was carried out for the detection of ESBL encoding genes (*bla* SHV, *bla* TEM, *bla* CTX-M, and *bla* OXA) [23]. The same isolates were tested for *bla* CMY-2 [24], which encodes pAmpC β-lactamase.

### Sequencing of β-lactamase TEM and SHV genes

To identify the identities of the β-lactamase TEM and SHV genes detected in the PCR assay, partial DNA sequence analysis of PCR amplicons for one representative ESBL-producing *E. coli* strain was performed. Amplified PCR products were purified using a QIAquick PCR purification kit (Qiagen, Germany) according to the manufacturer's instructions. Afterwards, sequencing was carried out using a BigDye V3.1 sequencing kit (Applied Biosystems, ThermoFisher Scientific, USA) with an ABI 3500 Genetic Analyzer (Applied Biosystems, ThermoFisher Scientific, USA).

### Nucleotide sequence accession numbers

The accession numbers of *E. coli bla* TEM and *bla* SHV gene sequences deposited in the GenBank are PP723879 and PP746544, respectively.

### Molecular detection of genes associated with diarrheagenic *E. coli* pathotypes

All ESBL-/pAmpC-producing *E. coli* strains were investigated to identify the diarrheagenic *E. coli* pathotypes via multiplex PCR targeting six virulence genes (*eaeA*, *bfpA*, *stx1*, *stx2*, *st*, and *lt*) [25]. The PCR mixture was preheated at 94 °C for 4 min followed by 30 cycles of denaturation, annealing, and extension at 94 °C for 30 s, 55 °C for 30 s, and 72°C for 1 min, respectively, then a final extension at 72°C for 10 min.

### PCR amplification of STa and STb classes in enterotoxigenic *E. coli* (ETEC) strains

ETEC isolates carrying heat-stable enterotoxin (ST) were subjected to PCR using oligonucleotide primers that encode STa and STb subunits. The following primers (5′-TCC GTG AAA CAA CAT GAC GG-3′ and 5′-ATA ACA TCC AGC ACA GGC AG-3′) and (5′-GCC TAT GCA TCT ACA CAA TC-3′ and 5′-TGA GAA ATG GAC AAT GTC CG-3′) (Metabion, Germany) were designed to amplify STa and STb, respectively [26]. The PCR reaction for each gene was performed in a 25 μL final volume containing 1 μL of each primer, 12.5 μL of Cosmo PCR red master mix (Willowfor, UK), 3 μL of DNA template, and completed up to 25 μL with nuclease-free water. The PCR cycling conditions were as follows: Initial denaturation at 94 °C for 5 min followed by 40 cycles of denaturation at 94 °C for 30 s, annealing at 52 °C and 51 °C for STa and STb, respectively, for 30 s, and extension at 72 °C for 30 s, then a final extension step at 72 °C for 5 min. The PCR amplicons were separated by agarose gel electrophoresis (BioRad, USA) at 100 V for 1 h and visualized under ultraviolet light after staining with ethidium bromide (2 μg/mL) (Sigma-Aldrich, USA), where a specific band of the STb gene was shown at 279 bp.

### Sequencing of ETEC STb gene and phylogenetic analysis

**Fig. 1:**
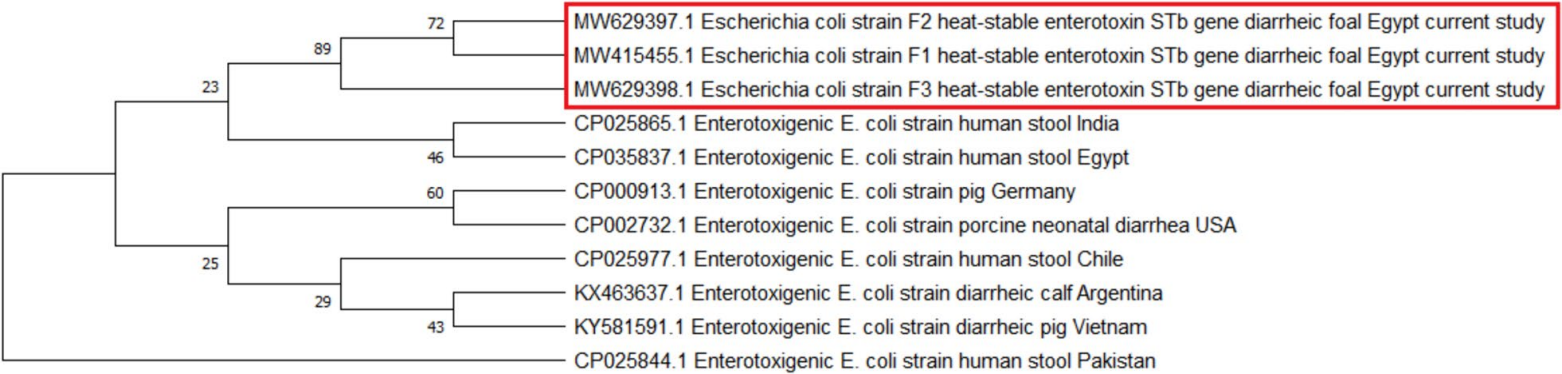
Phylogenetic tree of the STb gene of enterotoxigenic *E. coli* isolated from diarrheic foals. Phylogenetic bootstrap consensus tree was inferred via neighbor-joining approach using MEGA 7 software to show the evolutionary history and genetic relatedness between enterotoxigenic *E. coli* STb gene partial sequences obtained in this study and ETEC strains retrieved from GenBank records

The purified PCR products of the STb gene of three selected ESBL-pAmpC-producing ETEC isolates were subjected to partial sequencing using the Big Dye Terminator V3.1 sequencing kit (Applied Biosystems, ThermoFisher Scientific, USA) in an ABI 3500 Genetic Analyzer (Applied Biosystems, ThermoFisher Scientific, USA) according to the manufacturer's protocol. The obtained sequences were aligned against other ETEC strains retrieved from animals and humans available on GenBank to determine the public health significance of our isolates. A phylogenetic tree was constructed via the neighbor-joining approach with 500 replicates of the bootstrap consensus tree using MEGA 7 software (Fig. 1).

### Nucleotide sequence accession numbers

In the current study, the partial STb sequences of ETEC isolates obtained from diarrheic foals were deposited in the GenBank under the following accession numbers: MW415455, MW629397, and MW629398.

### Antibiotic sensitivity testing of ESBL-/pAmpC-producing ETEC isolates

Twenty-one ETEC strains were examined for susceptibility to antimicrobial agents (HiMedia, India) using the disk diffusion method according to the recommendations of CLSI [16]. The antibiotics used were: Ampicillin (AMP), cefotaxime (CTX), ceftazidime (CAZ), cefazolin (CZ), cefoxitin (CX), cefepime (CPM), ceftriaxone (CTR), cefpodoxime (CPD), aztreonam (AT), imipenem (IPM), meropenem (MRP), gentamicin (GEN), amikacin (AK), azithromycin (AZM), tetracycline (TE), doxycycline (DO), ciprofloxacin (CIP), norfloxacin (NX), trimethoprim-sulfamethoxazole (COT), chloramphenicol (C), and nitrofurantoin (NIT). Multidrug-resistant ETEC isolates were identified by resistance to at least one agent in three or more antimicrobial categories [27].

### Statistical analysis

The modified Wald method was utilized to calculate the 95% confidence interval (CI) of an overall prevalence value using the GraphPad QuickCalc online tool https://www.graphpad.com/quickcalcs/confInterval1/.

## Results

**Fig. 2:**
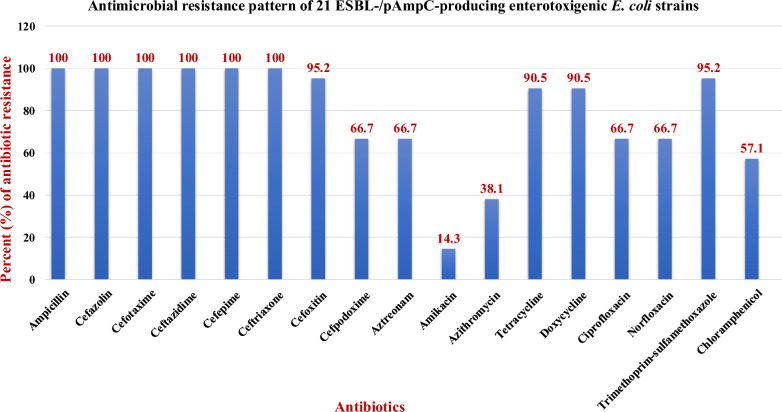
Antimicrobial resistance pattern of 21 ESBL-/pAmpC-producing enterotoxigenic *E. coli* strains

Out of 80 diarrheic foals, 26 (32.5%; 95% CI 23.21–43.39) were carriers of ESBL-/pAmpC-producing *E. coli*, with ESBL-producing *E. coli* isolates detected in 14 (17.5%) animals, and 12 (15%) animals carrying ESBL-pAmpC-producing *E. coli* (Table 1). Regarding ESBL-encoding genes, *bla* TEM and *bla* CTX-M were the most predominant ones (100%), followed by *bla* SHV (38.5%) and *bla* OXA (7.7%). Additionally, pAmpC (*bla* CMY-2) was co-harbored with ESBL encoding genes in 12 *E. coli* strains, as illustrated in Table 2. β-lactamase TEM and SHV genes of one representative *E. coli* isolate were sequenced and identified as belonging to ESBL TEM-52 and ESBL SHV-12, respectively. Twenty-one (80.8%) out of 26 isolates possessed the heat-stable enterotoxin (ST) gene, defining them as enterotoxigenic *E. coli* (ETEC), while other *E. coli* pathotypes could not be detected. Further characterization of the ST gene revealed that 1, 4, and 16 ETEC isolates were positive for STa, STb, and STa + STb, respectively (Table 2). The antimicrobial susceptibility pattern of the 21 ESBL-/pAmpC-producing ETEC is presented in Fig. 2, where all strains exhibited multi-drug resistance.

**Table 1:** Prevalence of ESBL-/pAmpC-producing *E. coli* among diarrheic foals

*E. coli* isolates	No. of examined animals	Positive animals	
		No.	%
ESBL-producing *E. coli*		14	17.5
ESBL-pAmpC-producing *E. coli*	80	12	15
Total		26	32.5

**Table 2:** Detection of β-lactamase encoding genes and ST gene among ESBL-/pAmpC-producing *E. coli* isolates

No. of isolates	ESBL encoding genes				pAmpC (*bla* CMY-2))	Heat stable enterotoxin (ST)
	*bla* SHV No. (%)	*bla* TEM No. (%)	*bla* CTX-M No. (%)	*bla* OXA No. (%)	No. (%)	No. (%)
4	–	+	+	–	–	–
4	–	+	+	–	–	STa + STb
4	–	+	+	–	+	STa + STb
4	+	+	+	–	+	STa + STb
3	+	+	+	–	–	STa + STb
2	+	+	+	–	+	STb
1	+	+	+	–	–	–
1	–	+	+	–	–	STb
1	–	+	+	+	–	STa
1	–	+	+	–	+	STb
1	–	+	+	+	+	STa + STb
Total 26	10 (38.5%)	26 (100%)	26 (100%)	2 (7.7%)	12 (46.2%)	21 (80.8%)

Resistance against ampicillin, cefotaxime, ceftazidime, cefazolin, cefepime, and ceftriaxone was identified in 100% (95% CI 81.76–100) of isolates (21/21), followed by cefoxitin and trimethoprim-sulfamethoxazole resistance in 20 (95.2%; 95% CI 75.58–99.99) isolates. Additionally, 19 (90.5%; 95% CI 69.88–98.55) strains were resistant to tetracyclines (TE, DO), and 14 (66.7%; 95% CI 45.22–82.95) isolates were resistant to cefpodoxime, aztreonam, and quinolones (CIP, NX), while resistance to chloramphenicol, azithromycin, and amikacin was observed in 12 (57.1%; 95% CI 36.52–75.56), 8 (38.1%; 95% CI 20.68–59.20), and 3 (14.3%; 95% CI 4.14–35.48) ETEC strains, respectively (Fig. 2). On the other hand, all isolates were susceptible to imipenem, meropenem, gentamicin, and nitrofurantoin.

## Discussion

Overall, 32.5% of diarrheic foals were positive for ESBL-/pAmpC-producing *E. coli*, with ESBL-producing *E. coli* isolates detected in 17.5%. This prevalence of ESBL-producing *E. coli* was higher than that reported in Germany (10.1%) [28] and the United States (6.3%) [29]; however, it was lower than that detected in the Netherlands (84%) [30], Ireland (65%) [16], the United Kingdom (28.7% and 50% in the 2008 and 2017 cohorts, respectively) [31], and the Czech Republic (32%) [32]. The differences in prevalence from those previously recorded may be due to regional variations, sampling facilities, management conditions, hygienic measures, and different antibiotic use practices. Regarding the detection of ESBL-encoding genes, the predominant ones were *bla* TEM (100%) and *bla* CTX-M (100%), as CTX-M enzymes mostly co-exist with TEM β-lactamases in bacteria of animal origin [33]. CTX-M was the most commonly detected ESBL type among *E. coli* isolates in equines [9, 34], while *bla* TEM was the most prevalent ESBL gene in a study concerning diarrheic foals [16]. Additionally, 15% of the examined foals harbored ESBL and pAmpC (*bla* CMY-2)-producing *E. coli* strains. CMY2 is the most prominent pAmpC gene among *E. coli* isolates, which can be transmitted between humans and animals [35]. It is associated with multi-drug resistance because of carrying resistance determinants for other antimicrobial agents on the same plasmids [36], leading to serious challenges with limited therapeutic options [37]. As a result, our findings indicate that feces of diarrheic foals may act as a vehicle for ESBL-/pAmpC-producing *E. coli*, implying that this infection can be transmitted between humans and horses [32, 38]. This poses a public health threat to human contacts, such as veterinarians, caretakers, and owners.

The investigation of virulence genes related to diarrheagenic *E. coli* pathotypes revealed that 21 (80.8%) out of 26 ESBL-/pAmpC-producing *E. coli* strains encoded a heat-stable enterotoxin (ST), which is produced by ETEC [1]. In contrast, other *E. coli* pathotypes could not be detected. To the best of our knowledge, this is the first study focusing on the occurrence of ESBL-/pAmpC-producing enterotoxigenic *E. coli* in diarrheic foals. Among animals, ETEC is mainly associated with newborn calves and piglets suffering from diarrhea [39, 40], but the prevalence of such a pathotype in foals is rare [40, 41]. In an earlier investigation [42], although diarrhea could not be induced in six foals with a K88 + STb + LT + ETEC strain isolated from a foal with diarrhea, two of the foals developed acute ulcerative gastritis, and another two had acute neutrophilic enteritis. The exact role of ETEC in foal diarrhea and its detrimental impact on the health of foals are yet to be elucidated. On the other hand, ETEC is the primary cause of traveler's diarrhea. It is more prevalent among children in developing countries [43], accounting for 75 million diarrheal episodes in children under 5 years of age [44]. In Egypt, ETEC is one of the leading causes of neonatal calf diarrhea [45–47], and it is a significant cause of diarrhea in children [48, 49]; however, the prevalence of ETEC among diarrheic foals in Egypt is unknown. ETEC is known to be transmitted via ingestion of contaminated food and water [1], and environmental contamination plays a role in transmitting ETEC to animals through the oral route [40, 50]. The predominance of ETEC among ESBL-/pAmpC-producing strains isolated from diarrheic foals in this study suggests that such animals could potentially be a source of ESBL-/pAmpC-producing ETEC infection in humans. However, whether water or soil contaminated with human feces is a source of ETEC infection in foals remains to be investigated.

ETEC encodes either heat-stable (ST) or heat-labile (LT) enterotoxins or both [1]. Significantly, ETEC isolates carrying ST cause more severe human illness than isolates harboring LT [43]. ST-producing ETEC has been associated with severe infantile diarrhea in Egypt [51] and Bangladesh [52]. Because STs have two independent subunits, STa and STb, that vary in structure and mode of action [26], further characterization of ST-producing ETEC was conducted in the present study in which one, four, and 16 isolates were positive for STa, STb, and STa + STb, respectively. The prevalence of STb was higher than that documented in the previous study [18], where the STb gene was found in one out of 61 *E. coli* isolates recovered from diarrheic foals. This result might be attributed to the fact that the STb gene is highly conserved among ETEC strains worldwide [53]. The presence of the STb gene is significant in the differentiation between commensal *E. coli* strains and those causing diarrhea [54], as it has been reported in diarrheic human isolates [55, 56]. Accordingly, in this study, partial sequencing of the STb gene of three selected ESBL-pAmpC-producing ETEC isolates was carried out. A phylogenetic tree was constructed to include ETEC strains from humans and animals (pigs and calves). The analysis revealed two clusters. The first cluster included STb sequences obtained from diarrheic foals in this work and those of ETEC retrieved from humans in India and Egypt. The second cluster comprised calf and pig ETEC isolates and a human strain from Chile. This finding highlights that intimate contact between humans and foals may allow the transmission of such strains to humans, representing a severe zoonotic risk. Therefore, hygienic measures should be implemented while handling infected animals and disposing of animal waste to avoid contamination of food, water and environment.

In veterinary and human medicine, multi-drug resistant ESBL-producing bacteria pose significant therapeutic challenges, especially in treating hospitalized and community infections [57, 58]. In the current investigation, twenty-one ESBL-/pAmpC-producing ETEC strains showed multi-drug resistance to critical antibiotics prescribed for human and equine medicine. In Egypt, ESBL-producing *E. coli* has been observed in 69.6% and 54.5% of patients with urinary tract infections and bloodstream infections, respectively [59, 60]. Also, 62.5% of *E. coli* isolates retrieved from various clinical specimens from different hospitals in Mansoura were ESBL producers [61]. This indicates that the extensive use of third-generation cephalosporins as empirical therapy in Egypt is suspected as the cause of ESBL-producing *E. coli* [59].

Moreover, *E. coli* strains that encode ST are more likely to be antibiotic-resistant than LT or LT and ST-producing isolates [43]. Additionally, antibiotic resistance and enterotoxin production genes are transferred together on a single plasmid [62, 63], indicating that the widespread use of antibiotics could lead to more dissemination of multidrug-resistant ETEC in humans and animals [64]. Multiple enteric Gram-negative pathogens exhibiting antimicrobial resistance, such as *E. coli*, *Proteus mirabilis*, *Proteus vulgaris*, *Enterobacter aerogenes*, *Citrobacter diversus*, and *Salmonella enterica* could be detected among diarrheic foals in Egypt [65]. This underscores the importance of developing new antimicrobial agents to reduce the emergence of antibiotic resistance as a threat to human health [66, 67]. Furthermore, an active surveillance policy should focus on antimicrobial resistance shedding and infection in foals [17] under the umbrella of the One Health concept, which promotes multidisciplinary collaboration between the environment and aspects of human and animal health [68]. Identifying risk factors associated with multidrug-resistance carriage in foals is necessary to minimize antimicrobial resistance in such animals. Ultimately, effective mitigation strategies should be implemented, such as farm management, biosecurity, and hygiene [69].

## Conclusion

The occurrence of ESBL-/pAmpC-producing ETEC among diarrheic foals highlights horses as a possible zoonotic reservoir for such strains. Further studies should focus on the sources of ESBL-/pAmpC-producing ETEC infection in foals, describing its molecular characteristics and pathogenicity in foals to limit transmission between horses, humans and the environment. Moreover, determining risk factors related to the shedding of ESBL-/pAmpC-producing ETEC in diarrheic foals could help equine veterinarians manage infection. Considering the present situation in veterinary medicine, it is clear that more emphasis on effective management and hygienic measures in equine farms is crucial to prevent such infections in foals.

## Data Availability

All data generated or analyzed during this study are included in this published article.
